# Comparison of Unilateral and Bilateral Jump Training on Physical Performance Adaptations in Prepubertal and Pubertal Youth Soccer Players

**DOI:** 10.3390/jfmk11020146

**Published:** 2026-04-01

**Authors:** Wajdi Dardouri, Raouf Hammami, Abdelkader Mahmoudi, Roland van den Tillaar

**Affiliations:** 1 Department of Sport Sciences and Physical Activity, College of Education, University of Hail, Hail 81411, Saudi Arabia; wajdi.dardouri@gmail.com; 2 University Campus of Manouba, Higher Institute of Sport and Physical Education of Ksar-Said, Manouba 2010, Tunisia; raouf.cnmss@gmail.com; 3 Tunisian Research Laboratory ‘Sports Performance Optimization’, National Center of Medicine and Science in Sports, (CNMSS-LR09SEP01), Tunis 1004, Tunisia; abdelkadermahmoudi.cnmss@gmail.com; 4 Department of Sports Sciences and Physical Education, Nord University, 7600 Levanger, Norway

**Keywords:** plyometrics, fundamental movement skills, training specificity, stretch–shortening cycle, maturation

## Abstract

**Objective:** This randomized controlled trial aimed to investigate how volume-matched unilateral and bilateral jump training affects physical performance in prepubertal and pubertal male youth soccer players and to examine whether maturational status influences these training adaptations. **Methods:** Sixty-five male soccer players (age 10.5 ± 2.9 years; height 136.7 ± 17.8 cm; body mass 32.8 ± 8.6 kg; maturity offset −1.6 ± 1.0 years) completed an 8-week training program (two sessions/week). Participants were randomly assigned to a bilateral jump group (*n* = 22), unilateral jump group (*n* = 22), or control group (*n* = 21). Performance was evaluated in a single testing session, which included horizontal jump tests (bilateral standing long jump and single-leg hop distance), linear sprint tests over 10 m (acceleration) and 30 m (maximal sprint performance) using timed trials, and change-of-direction (COD) ability assessed via a standardized timed COD test. **Results:** Significant main effects of time, maturation, and time × group interactions were observed for all outcomes (*p* ≤ 0.013). A maturation × group interaction was found for bilateral jump performance (*p* = 0.045), a group effect for 10 m sprint time (*p* = 0.015), and a time × maturation × group interaction for COD performance (*p* < 0.001). Both training groups had improved jump performance (jump distance) and 10 m sprint time across maturity levels, while no changes were observed in the control group. For 30 m sprint time, improvements were observed in both training groups in prepubertal players, whereas only the unilateral group showed improvements in pubertal players. COD performance (completion time) improved in the unilateral group at both maturity levels and in the bilateral group at the pubertal level. **Conclusions:** Structured jump training enhances horizontal jump distance, sprint performance, and COD ability in youth soccer players. Adaptations appear to be influenced by training modality and maturation, although these effects may vary depending on the specific performance task.

## 1. Introduction

The concept of the transfer of training refers to the extent to which performance improvements in one motor task lead to corresponding enhancements in another task that shares similar mechanical, neural, or coordinative demands [1,2]. In youth athletic development, such transfer is often explained through shared neuromuscular and biomechanical characteristics across tasks, rather than through isolated skill acquisition. Sprint acceleration, horizontal jumping, and change-of-direction (COD) performance, for example, rely on common underlying capacities, including rapid force production, effective force orientation, intermuscular coordination, and stretch–shortening cycle function [3,4,5]. From this perspective, these tasks can be viewed as different expressions of lower-limb neuromuscular performance that are highly relevant to sport contexts such as soccer.

Although these performance qualities are sometimes discussed within the broader framework of fundamental movement skills (FMSs), in trained youth populations, they are more appropriately interpreted as sport-related neuromuscular capacities that underpin athletic performance. Sprinting, unilateral hopping, and bilateral jumping share key mechanical features, particularly the ability to generate and direct force efficiently in the horizontal and vertical directions [4,6]. Similarly, COD performance requires rapid deceleration and re-acceleration, placing substantial demands on eccentric strength, dynamic stability, and coordinative control [7,8]. Therefore, examining how training interventions transfer across these tasks provides insight into the specificity of neuromuscular adaptations in youth athletes.

Plyometric training is widely recognized as an effective method for improving explosive neuromuscular performance in youth, with consistent evidence demonstrating enhancements in jumping ability, sprint speed, and COD performance [7,8,9]. However, the extent to which these improvements transfer across tasks may depend on the specific characteristics of the training stimulus. In particular, the distinction between unilateral and bilateral exercise modalities has received increasing attention. Unilateral exercises impose greater demands on balance, stabilization, and interlimb coordination, which may enhance transfer to tasks such as sprinting and COD that are inherently unilateral in nature [10,11]. In contrast, bilateral exercises may facilitate greater overall force production due to higher loading capacity and reduced stability constraints [12]. These differences suggest that unilateral and bilateral plyometric training may induce distinct neuromuscular adaptations, with potentially different implications for sport-specific performance.

Despite these theoretical considerations, empirical evidence comparing unilateral and bilateral plyometric training in youth remains limited. Notably, Drouzas, et al. [13] conducted a randomized controlled trial in preadolescent soccer players and reported greater improvements in short-sprint performance, strength, and power following unilateral plyometric training compared with a control group. While these findings support the effectiveness of unilateral training, direct comparisons between unilateral and bilateral modalities are scarce, and the relative transfer of these approaches to sprint and COD performance remains unclear. Moreover, existing studies have largely focused on single age groups, limiting the understanding of how developmental factors may influence training adaptations.

Maturational status represents a key factor that may modulate responsiveness to training in youth athletes. During late childhood (approximately 6–12 years), ongoing neural development and high levels of plasticity may facilitate rapid improvements in coordination and neuromuscular function [14,15]. In contrast, the adolescent growth spurt is often associated with temporary disruptions in coordination and movement efficiency, commonly referred to as “adolescent awkwardness” [16]. These maturational changes may influence both the magnitude and specificity of adaptations to plyometric training, as well as the transfer of these adaptations to performance tasks such as sprinting and COD.

Taken together, current evidence supports the effectiveness of plyometric training for enhancing neuromuscular performance in youth athletes; however, important gaps remain. Specifically, it is unclear whether unilateral and bilateral plyometric training differentially transfer to sprint and COD performance and whether these effects are moderated by maturational status. Addressing these questions is essential for refining training strategies and optimizing performance development in youth soccer.

Therefore, the present randomized controlled trial had two primary aims. First, it sought to compare the effects of volume-matched unilateral and bilateral jump training programs on measures of physical performance, including jumping, sprinting, and change-of-direction ability, in child and adolescent soccer players. It was hypothesized that both training modalities would improve performance outcomes but that adaptations would demonstrate task specificity, with unilateral training producing greater improvements in sprinting and COD performance due to its higher movement specificity. Second, an exploratory subgroup analysis examined whether maturational status influenced adaptive responses to the respective training interventions. No a priori hypotheses were formulated for this exploratory analysis.

## 2. Methods

### 2.1. Experimental Design and Methodology

**Figure 1:**
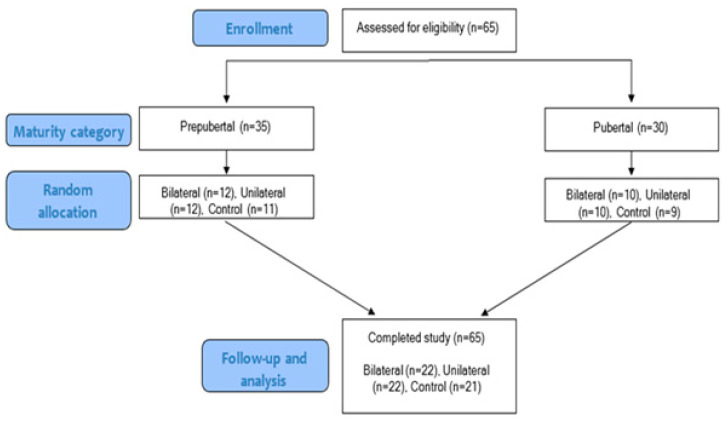
Study design. Arrows indicate the progression of participants through the study phases, including enrollment, maturity classification, random allocation, and follow-up/analysis. Blue boxes represent study phases (enrollment, maturity category, random allocation, and follow-up/analysis), while white boxes indicate participant groups and sample sizes at each stage. Participants were classified as prepubertal (*n* = 35) or pubertal (*n* = 30), then randomly assigned to bilateral, unilateral, or control groups. All participants completed the study (*n* = 65).

This randomized controlled trial examined the effects of an 8-week, twice-weekly unilateral or bilateral jump training program on jumping performance, sprint speed, and change-of-direction (COD) ability in male youth soccer players. Participants were randomly assigned to a bilateral jump group (*n* = 22), unilateral jump group (*n* = 22), or control group (*n* = 21) using a stratified block randomization procedure, with blocks of six to ensure balanced group sizes (Figure 1). Stratification was performed based on maturational status to achieve an even distribution of prepubertal and pubertal participants across groups. The randomization sequence was computer-generated by a researcher not involved in testing or training; however, allocation concealment was not implemented, and group assignments were known at the time of allocation. Outcome assessors and data analysts were blinded to group assignment; participant and trainer blinding was not feasible due to the nature of the intervention. All participants completed two familiarization sessions two weeks prior to baseline testing. A qualified strength and conditioning specialist supervised all procedures.

### 2.2. Participants

A priori power analysis was conducted with reference to the study of Ramirez-Campillo, et al. [17] on plyometric jump training in adolescent soccer players. The analysis indicated that eight participants per group would provide 80% statistical power (β = 0.20) at an alpha level of 0.05, assuming an effect size of 0.20. Sixty-five healthy male soccer players (age 10.5 ± 2.9 years; height 136.7 ± 17.8 cm; body mass 32.8 ± 8.6 kg; maturity offset −1.6 ± 1.0 years) volunteered to participate. Players were members of a soccer academy in Takelsa, Tunisia, with 2–3.5 years of structured training (~3 sessions/week). Written informed consent and assent were obtained from legal guardians and participants. Exclusion criteria included current musculoskeletal injuries, any medical condition contraindicating high-intensity exercise, participation in structured plyometric training within the previous six months, or failure to obtain parental consent. This study was approved by the ethics committee of the National Centre of Medicine and Science of Sports, Tunis (CNMSS-LR09SEP01), and conducted in accordance with the Declaration of Helsinki. Participants with musculoskeletal, neurological, or orthopedic disorders within six months prior to this study were excluded.

### 2.3. Maturational Assessment and Anthropometry

Height was measured using a stadiometer, body mass with a digital scale, and skinfold thickness at biceps, triceps, subscapular, and suprailiac sites. Body fat percentage was estimated according to Deurenberg, et al. [18]. Maturity offset was calculated using Moore, et al. [19] predictive equations to estimate years from peak height velocity. Players were classified as either prepubertal or pubertal based on their maturity offset values: those with a maturity offset between −3 and −1 were considered prepubertal, whereas those with a maturity offset between −1 and 1 were classified as pubertal. We acknowledge that maturity offset is an indirect estimate and that this binary classification does not fully capture the continuous nature of maturation; therefore, interpretations related to maturational status are made cautiously.

### 2.4. Testing Procedure

One week prior to the intervention, participants attended a familiarization session to become acquainted with all tests and training exercises. During this session, proper techniques for unilateral and bilateral jump exercises were demonstrated and practiced. Participants were instructed to maintain their usual nutrition and sleep routines throughout this study. All testing followed the same sequence for pre- and post-intervention assessments. The testing protocol included: (a) body composition evaluation, (b) bilateral and unilateral horizontal jump performance, (c) sprint assessments over 10 m and 30 m, and (d) change-of-direction (COD) ability tests. Before each testing session, participants completed a standardized 10 min warm-up, consisting of submaximal running (e.g., skipping, hip in-and-out drills), balance exercises (e.g., forward and backward beam walking, single-leg stance on unstable surfaces), and neuromuscular activation drills (e.g., dynamic and isometric squats, single-leg deep squats). Each performance test was separated by 5–10 min of rest to minimize fatigue. For all jump, sprint, and COD tests, participants performed two maximal attempts, with three minutes of rest between attempts. The best score from the two trials was used for further statistical analyses. All testing and training sessions were supervised by certified coaches to ensure correct technique and participant safety.

#### 2.4.1. Sprint Performance

Running times were assessed during a 30 m sprint test, with split times recorded at 10 m and 30 m. Participants began from a stationary standing position 20 cm behind the first timing gate, with one foot positioned at the start line. No auditory start signal was given to eliminate reaction time influence. Times were recorded using photocell timing gates (Brower Timing Systems, Salt Lake City, UT, USA; accuracy 0.01 s) positioned 0.4 m above ground [20,21]. This protocol demonstrated excellent reliability (ICC = 0.99) [22].

#### 2.4.2. Change-of-Direction Performance

**Figure 2:**
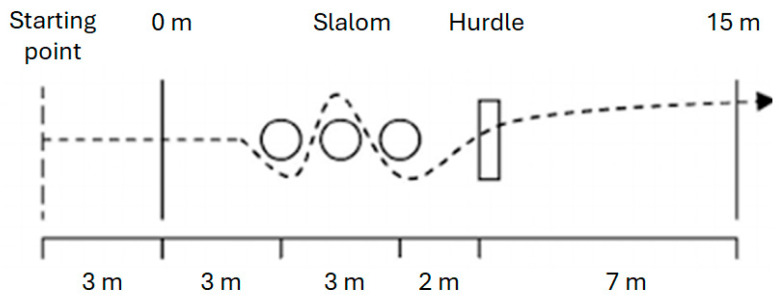
Schematic diagram of agility test course. The dashed line with arrows indicates the running trajectory followed by the participants during the test. Solid vertical lines represent timing gates (start, 0 m, and 15 m). Circles indicate slalom poles, and the rectangle represents the hurdle obstacle. Distances between elements are shown along the bottom of the figure.

The 15 m COD test required participants to run from 3 m behind the start line, complete a 3 m straight sprint, navigate a 3 m slalom (three poles, 1.5 m apart), clear a 0.5 m hurdle placed 2 m beyond the final pole, and sprint 7 m to the finish gates [23] (Figure 2). This test demonstrated excellent reliability (ICC = 0.94) [24].

#### 2.4.3. Horizontal Jump Performance

The horizontal jump tests (bilateral and unilateral) followed the protocol of Ramírez-Campillo, et al. [25]. Participants performed maximal horizontal jumps using either both legs (bilateral) or a single-leg stance (right and left). Participants performed a standing long jump using a bilateral stance behind a starting line [26]. Arm swing was permitted. Jump distance was measured from take-off to heel contact on landing using a metal tape. Reliability was reported as excellent (ICC = 0.91; range 0.83–0.96) [27].

In both bilateral and unilateral jumps, participants used an arm swing and approximately 120° knee flexion prior to take-off. Two trials were completed for each condition in randomized order. The best performance for the bilateral jump and the best performance from each leg in the unilateral jump were recorded, with unilateral scores averaged for analysis. Similar horizontal jump tests demonstrated very high reliability (ICC = 0.96; range 0.90–0.99) [28].

### 2.5. Training Intervention

Following the approach of Bogdanis, et al. [29], the bilateral group performed all exercises using both legs simultaneously. The training protocols for each group included session frequency, duration, exercise type, and contact-based volume. To align training volume between conditions, the unilateral group performed half the repetitions per leg. Training volume was operationally defined as the total number of ground contacts across both limbs per session, rather than simply the number of repetitions. Repetitions per leg in the unilateral group were reduced to match the total number of ground contacts performed in bilateral conditions. This approach was used to standardize contact-based exposure between groups, as each jump represents a discrete interaction with the ground (Table 1). We acknowledge that matching ground contacts reflects an operational definition of training volume and contact-based exposure and does not necessarily indicate equivalence in mechanical loading, work, or internal training load between conditions.

Session duration, exercise selection, and rest intervals were matched between groups to ensure comparable training structure. Both the unilateral and bilateral groups completed two training sessions per week, each lasting approximately 30 min. The control group also participated in two weekly sessions, maintaining their regular 90 min soccer training sessions that included technical drills, tactical exercises, and small-sided games (Table 2).

### 2.6. Statistics

Statistical analyses were conducted using JASP 095.1.0 (University of Amsterdam, Amsterdam, the Netherlands). Data normality and homogeneity of variance were assessed using the Shapiro–Wilk and Levene tests, respectively. A one-way analysis of variance (ANOVA) was done on the groups to identify eventual differences at baseline for each parameter. A 2 (test occasion: pre and post; repeated measures) × 2 (maturity: pre- and pubertal) × 3 (training group: bilateral, unilateral and control groups) analysis of variance was performed for each parameter. In addition, differences in performance from pre- to post-test were calculated for each group at each maturity level, and a 2 (maturity: pre- and pubertal) × 3 (training group: bilateral, unilateral and control groups) ANOVA was performed on each parameter to get a better understanding of the changes per group and maturity level. When significant effects were found, Holm–Bonferroni adjustments were applied to post hoc comparisons within each outcome to locate the differences, and no additional adjustment across outcome variables was performed, as each outcome was analyzed separately. Effect size was evaluated with Eta partial squared where 0.01 < η^2^ < 0.06 constitutes a small effect, a medium effect when 0.06 < η^2^ < 0.14 and a large effect when η^2^ > 0.14 [30].

## 3. Results

All data was normally distributed. No significant difference for any of the parameters was found between the groups at baseline (*p* ≥ 0.05). Significant effects of time (F ≥ 6.52, *p* ≤ 0.013, η^2^ ≥ 0.10), maturation (F ≥ 6.73, *p* ≤ 0.012, η^2^ ≥ 0.10), and time x group interaction (F ≥ 4.85, *p* ≤ 0.011, η^2^ ≥ 0.14) were seen for all tested parameters. In addition, a significant maturation × group interaction was found for the bilateral jumps (F = 3.26, *p* = 0.045, η^2^ = 0.10), a group effect with the 10 m sprint times (F = 4.55, *p* = 0.015, η^2^ = 0.13) and a time × maturation × group interaction for the COD test (F = 8.45, *p* < 0.001, η^2^ = 0.123).

**Figure 3:**
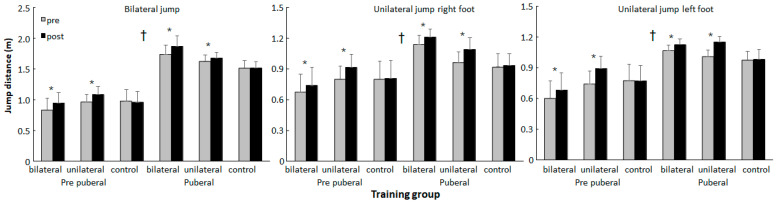
The mean (95% CI) bilateral and unilateral jump performance per group and maturity level at pre- and post-test. * indicates a significant change from pre- to post-test. † indicates a significant difference for all groups between the two maturity levels at pre- and post-test.

**Figure 4:**
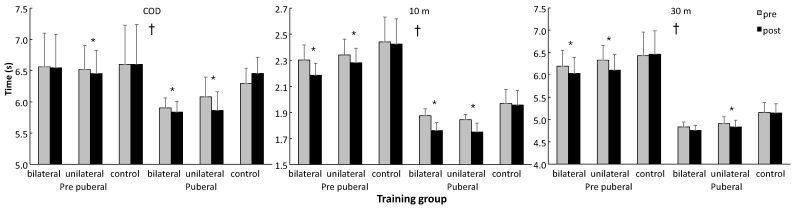
The mean (95% CI) change-of-direction (COD), 10 m and 30 m sprint performance per group and maturity level at pre- and post-test. * indicates a significant change from pre- to post-test. † indicates a significant difference for all groups between the two maturity levels at pre- and post-test.

Holm–Bonferroni post hoc comparisons revealed that the pubertal participants performed better in all parameters at pre- and post-tests compared with the prepubertal participants (Figure 3 and Figure 4) and that the control groups did not show significant changes in their performance between the tests, while for the jumps and 10 m sprint test, both intervention groups showed increased performance at both maturity levels (Figure 3). In the COD test, the unilateral group at both maturity levels did run faster at post-test, while the bilateral group of pubertal participants showed increased COD performance (Figure 4). In terms of the 30 m sprint, both the bi- and unilateral groups at the prepubertal maturity level showed increased performance, while at the pubertal level, only the unilateral group showed significantly increased 30 m performance (Figure 4).

**Figure 5:**
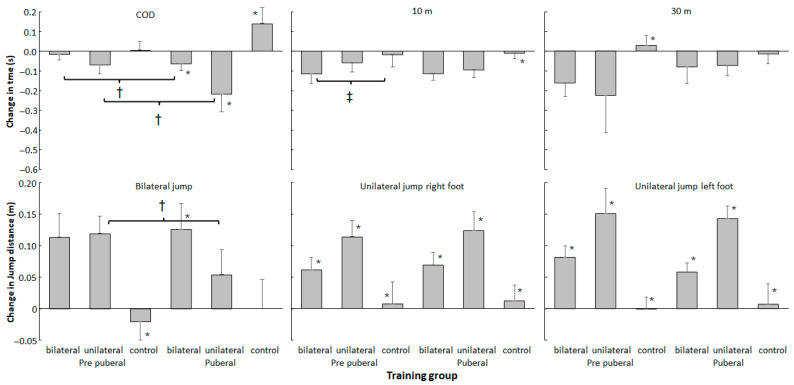
The mean (95% CI) change from pre- to post-test for each parameter per group and maturity level at pre- and post-test. * indicates a significant difference with the other two groups at this maturity level. † indicates a significant difference between these two groups at each maturity level. ‡ indicates a significant difference between these two groups for this maturity level.

In addition, Holm–Bonferroni post hoc comparisons showed that the increase in performance in bilateral jumps in the bilateral pubertal group was significantly higher than that in the other two groups at this maturity level, while for the unilateral group, the increase in jump performance was much higher in the prepubertal group compared with the pubertal group. In addition, the control group showed decreased performance at the prepubertal level, while the other two groups showed a significant increase (Figure 5). For the COD test at the pubertal level, every group had a significantly different change in performance, while both the bilateral and unilateral pubertal groups showed significantly increased COD performance compared to these groups at the prepubertal level (Figure 5). The significant group effect in the 10 m sprint was caused by the fact that both training groups showed increased sprint performance at both maturity levels, while the control group did not show significant changes in sprint performance, thereby indicating that the control group performed significantly worse when the maturity level and pre- and post-test were considered together (Figure 4 and Figure 5).

## 4. Discussion

The primary aim of this study was to compare the effects of volume-matched unilateral and bilateral jump training programs on physical performance in prepubertal and pubertal youth soccer players. We hypothesized that both training modalities would improve jump, sprint, and change-of-direction (COD) performance and that adaptations in jump performance would reflect training specificity. A secondary aim was to examine whether maturational status influenced these adaptations.

Consistent with our hypotheses, both unilateral and bilateral jump training produced significant improvements in jump performance and 10 m sprint times, while the control group showed no meaningful changes. These improvements were reflected in significant time and time × group interactions across performance measures. Although the underlying mechanisms were not directly measured in this study, the gains are likely related to neuromuscular adaptations such as enhanced motor unit recruitment, improved intermuscular coordination, and the more efficient use of the stretch–shortening cycle, which have been reported in previous plyometric training studies in youth [31].

Evidence of training specificity was apparent in the jump performance outcomes. For unilateral jump performance, improvements were generally greater following unilateral training at both maturity levels. For bilateral jump performance, prepubertal athletes improved similarly following both training modalities, whereas pubertal athletes showed larger gains in the bilateral group. A recent meta-analysis reported that unilateral plyometric training produces significantly larger effects on single-leg jumping ability compared with bilateral training, whereas bilateral protocols tend to be more effective for enhancing bilateral jump performance, consistent with the principle of task-specific adaptation in neuromuscular training (e.g., greater SMD for single-leg jumps after unilateral training) and greater bilateral force expression after bilateral training [32]. Pubertal athletes, who typically exhibit increased muscle mass and strength due to hormonal changes (e.g., testosterone), may be better able to generate and coordinate high bilateral force outputs, making bilateral training more effective at this stage [14,33]. In contrast, prepubertal athletes rely more on coordination and motor control, which can be stimulated effectively by both unilateral and bilateral exercises [34].

Regarding sprint performance, both training modalities improved 10 m sprint speed at both maturational levels, while the control group showed no change, indicating that jump-based plyometric training can enhance short-distance acceleration in youth athletes. Some studies in youth athletes combining plyometric and sprint or COD-specific drills have shown improvements in COD performance regardless of modality, highlighting that integrated approaches can also effectively enhance COD speed in adolescent populations [35]. For 30 m sprints, prepubertal participants improved similarly with both training types, whereas pubertal participants improved primarily with unilateral training. This may indicate that unilateral training better targets horizontal force production and unilateral coordination demands in longer sprints among more mature athletes, although direct measurements of horizontal force were not collected. These results partially support previous studies reporting that plyometric training enhances sprint performance in youth, with modality-specific effects depending on sprint distance and maturation [36,37].

COD performance also improved following training, with significant time × maturation × group interactions. Strength or plyometric training interventions in youth footballers have demonstrated improved COD at moderate angles regardless of training method, suggesting that both neuromuscular and strength adaptations contribute to COD improvements [38]. These findings underscore the complexity of COD development in youth and suggest that while unilateral plyometric training may confer particular advantages for coordination-intensive directional changes, modality-specific effects may interact with test angle, maturity status, and overall training context. Prepubertal athletes showed significant COD gains only after unilateral training, whereas pubertal athletes improved with both modalities but to a greater extent following unilateral training. These findings suggest that unilateral training may better support complex, multi-directional tasks, likely due to higher interlimb coordination and force application demands. This aligns with the prior literature highlighting the importance of unilateral plyometric exercises for enhancing agility and multi-directional performance in youth athletes [39,40], though the underlying neuromuscular mechanisms remain hypothetical, as neuromuscular activation patterns, tendon behavior, and muscle architecture were not directly assessed [41,42].

Across all performance measures, pubertal participants exhibited higher absolute values at both pre- and post-test compared with prepubertal participants, consistent with previous research showing that puberty is associated with greater strength and power due to hormonal increases, greater muscle mass, and enhanced neuromuscular coordination [41,42,43]. Meta-analytic evidence supports our findings, indicating that while all age groups benefit from plyometric training, pubertal athletes show larger absolute and relative improvements, likely reflecting maturational differences in strength and neuromuscular function [34]. Nevertheless, the present findings demonstrate that structured jump training can elicit meaningful performance improvements across all maturational stages, supporting the effectiveness of early neuromuscular interventions in youth athletes [44].

Overall, the results highlight the importance of considering both training specificity and maturational status when designing plyometric programs. While this study provides evidence for performance improvements following volume-matched unilateral and bilateral jump training, mechanistic explanations regarding neural adaptations and force production remain hypotheses that should be tested in future studies using direct measures such as EMG, tendon properties, or muscle architecture. The present findings have direct implications for youth soccer training. First, both unilateral and bilateral jump training can be safely and effectively incorporated to enhance jumping, sprinting, and COD performance. For prepubertal athletes, either modality may be used, as both provide meaningful neuromuscular stimulation. For pubertal athletes, bilateral training may be preferable to maximize bilateral force production and jumping performance, while unilateral training may be emphasized to enhance sprinting and COD performance. Coaches should implement two sessions per week, with volume matched to individual capabilities and technical proficiency, and ensure exercises are supervised by qualified personnel to maintain proper technique and reduce injury risk. Additionally, incorporating a combination of unilateral and bilateral exercises within the same program may optimize adaptations across multiple performance domains, supporting well-rounded athletic development in youth soccer players.

A limitation of the present study is the absence of mechanistic measures, such as electromyographic or biomechanical analyses, which restricts the understanding of the specific neuromuscular mechanisms driving the observed adaptations. Future research should incorporate these measures to clarify the neural and mechanical contributions to performance improvements following different jump training modalities in youth athletes. Importantly, even very young participants (mean age 8.1 years, some <6 years) achieved substantial gains despite less mature musculotendinous and neuromuscular characteristics, highlighting the high trainability of youth across maturational stages.

## 5. Conclusions

Structured jump training improved jumping, sprinting, and change-of-direction (COD) performance in both prepubertal and pubertal youth soccer players. Unilateral and bilateral training produced broadly similar improvements in jump performance, although some variation was observed across maturation groups: bilateral jump performance showed slightly greater gains in pubertal athletes in the bilateral group, while unilateral jump performance tended to improve more in prepubertal athletes in the unilateral group.

Sprint performance improved over 10 m in both training groups, whereas improvements over 30 m were observed primarily in prepubertal participants and, to a lesser extent, in pubertal athletes performing unilateral training. These findings suggest that short-distance acceleration is responsive to jump training across maturation stages, while adaptations in longer sprint distances may be more variable and potentially influenced by training modality. However, given the modest subgroup sample sizes and the absence of direct mechanical measures, these observations should be interpreted with caution.

For COD performance, improvements appeared to be influenced by both training modality and maturation status. Unilateral training was associated with gains across maturity levels, whereas bilateral training appeared to show slightly greater benefits in pubertal participants. The underlying mechanisms remain unclear and were not directly assessed.

From a practical perspective, both unilateral and bilateral jump exercises can be included to support jumping, sprint acceleration, and COD performance in youth soccer players. While some results suggest that training modality and maturation may influence outcomes, these findings are preliminary, and no firm modality-specific recommendations can be made. Practitioners are encouraged to adopt a balanced and flexible approach, combining different training exercises and considering individual athlete characteristics, while further research with larger samples is needed to clarify potential maturity-specific adaptations.

## Figures and Tables

**Table 1:** Bilateral and unilateral training programs.

Exercise	Sets	Bilateral Reps	Unilateral Reps per Leg	Total Foot Contacts (Bilateral)	Total Foot Contacts (Unilateral)	Rest (Between Sets)
20 cm Drop Jumps	3	6	3	18	18	1 min
Horizontal Jumps	3	10	5	30	30	1 min
Lateral Hops	3	10	5	30	30	1 min

**Table 2:** Summary of training protocols for bilateral jump, unilateral jump, and control groups.

Group	Session Frequency	Session Duration	Training Content	Training Volume
**Bilateral Jump**	2 sessions per week	~30 min	Bilateral plyometric exercises (both legs simultaneously)	Total ground contacts matched across both limbs
**Unilateral Jump**	2 sessions per week	~30 min	Unilateral plyometric exercises (one leg at a time)	Half repetitions per leg; total ground contacts matched to bilateral group
**Control**	2 sessions per week	90 min	Regular soccer training: technical drills, tactical exercises, small-sided games	No plyometric training; normal soccer training volume

## Data Availability

The original contributions presented in this study are included in the article/Supplementary Material. Further inquiries can be directed to the corresponding authors.

## Supplementary Materials

The following supporting information can be downloaded at: https://www.mdpi.com/article/10.3390/jfmk11020146/s1, Table S1: All statistical analyses.
