# Supplementary material for: Comparison of Unilateral and Bilateral Jump Training on Physical Performance Adaptations in Prepubertal and Pubertal Youth Soccer Players
Source: J Funct Morphol Kinesiol. 2026 Apr 1;11(2):146. doi: 10.3390/jfmk11020146 (PMC13108049; doi:10.3390/jfmk11020146)
Supplement: Supplementary file 1 [file jfmk-11-00146-s001.zip › jfmk-4220583-supplementary.pdf]

Table S1. All statistical analyses.

	Time			Time x maturation			Time x group			Time x group x maturation			Maturation			Group			maturation x group		
	F	P	η^2	F	P	η^2	F	P	η^2	F	P	η^2	F	P	η^2	F	P	η^2	F	P	η^2
Bilateral	67.2	< .001	0.53	0.44	0.51	0.01	23.8	< .001	0.45	2.98	0.06	0.09	127.1	< .001	0.68	1.1	0.33	0.04	3.26	0.05	0.10
Unilateral left	136.3	< .001	0.70	0.43	0.51	0.01	32.3	< .001	0.52	0.02	0.98	<.01	18.8	< .001	0.24	0.8	0.48	0.03	3.44	0.04	0.10
Unilateral right	177.3	< .001	0.75	0.51	0.48	0.01	56.8	< .001	0.66	0.68	0.51	0.02	35.5	< .001	0.38	1.0	0.37	0.03	2.15	0.13	0.07
CoD	6.5	0.013	0.10	0.43	0.52	0.01	18.5	< .001	0.39	8.46	< .001	0.23	6.7	0.01	0.10	1.0	0.38	0.03	0.55	0.58	0.02
10 m	51.4	< .001	0.47	0.22	0.64	0.00	9.2	< .001	0.24	0.50	0.61	0.02	93.1	< .001	0.61	4.6	0.02	0.13	0.28	0.76	0.01
30 m	16.7	< .001	0.22	2.19	0.15	0.04	4.9	0.01	0.14	1.72	0.19	0.06	93.6	< .001	0.61	2.2	0.12	0.07	0.01	0.99	<.01

Yellow indicates a significant effect on a $p < 0.05$ level
